# The influence of a maternal vegan diet on carnitine and vitamin B2 concentrations in human milk

**DOI:** 10.3389/fnut.2023.1107768

**Published:** 2023-08-04

**Authors:** Hannah G. Juncker, Chris H. P. van den Akker, Pauline L. Meerdink, Aniko Korosi, Frédéric M. Vaz, Johannes B. van Goudoever, Britt J. van Keulen

**Affiliations:** ^1^Department of Pediatrics, Amsterdam UMC, Vrije Universiteit, University of Amsterdam, Emma Children’s Hospital, Amsterdam, Netherlands; ^2^Amsterdam Reproduction and Development Research Institute, Amsterdam, Netherlands; ^3^Swammerdam Institute for Life Sciences—Center for Neuroscience, University of Amsterdam, Amsterdam, Netherlands; ^4^Department of Pediatrics—Neonatology, Amsterdam UMC Location University of Amsterdam, Amsterdam, Netherlands; ^5^Department of Clinical Chemistry and Pediatrics, Laboratory Genetic Metabolic Diseases, Emma Children's Hospital, Amsterdam UMC Location University of Amsterdam, Amsterdam, Netherlands; ^6^Amsterdam Gastroenterology Endocrinology Metabolism, Inborn Errors of Metabolism, Amsterdam, Netherlands; ^7^Core Facility Metabolomics, Amsterdam UMC, Amsterdam, Netherlands; ^8^United for Metabolic Diseases, Amsterdam, Netherlands

**Keywords:** breast milk, lactation, riboflavin, veganism, carnitine

## Abstract

**Background:**

The maternal diet greatly influences the nutritional composition of human milk. With the rise of vegan diets by lactating mothers, there are concerns about the nutritional adequacy of their milk. Two important nutrients, vitamin B2 and carnitine, are mostly ingested via animal products.

**Objective:**

We investigated the influence of a vegan diet on the vitamin B2 and carnitine concentrations in milk and serum of lactating women.

**Methods:**

In this case–control study, 25 lactating mothers following an exclusive vegan diet were comparted to 25 healthy lactating mothers with an omnivorous diet without use of supplements. High-performance liquid chromatography and liquid chromatography–tandem mass spectrometry were used to measure vitamin B2 and carnitine concentrations, respectively. A linear regression model was used to determine differences in human milk and serum concentrations between study groups.

**Results:**

Vitamin B2 concentrations in human milk and serum did not differ between study groups. While the human milk free carnitine (C_0_) and acetyl carnitine (C_2_) concentrations did not differ between study groups, serum carnitine concentrations were lower in participants following a vegan diet than in omnivorous women (*p* < 0.0001).

**Conclusion:**

A maternal vegan diet did not affect human milk concentration of vitamin B2 and carnitine. Breastfed infants of mothers following an exclusive vegan diet therefore are likely not at increased risk of developing a vitamin B2 or carnitine deficiency.

## 1. Introduction

Breastfeeding is the preferred modus of feeding for infants. The nutritional value of human milk is thought to be independent from subacute changes in maternal dietary intake. However, when the intake of certain nutrients is restricted persistently, a deficit of these nutrients in human milk may occur. With the global increase of specific diets such as veganism, concerns have been raised on the nutritional adequacy of milk from lactating mothers following such a diet.

In a vegan diet, all animal products are excluded from consumption. It is known that a vegan diet, if not properly planned, may result in a deficient intake and subsequently low serum concentrations of some nutrients, including vitamin B2, vitamin B12, zinc, selenium, iodine, carnitine, calcium, and omega-3 fatty acids (1–6). Furthermore, previous research in lactating mothers following a vegan diet has shown an altered human milk composition, for example changes in fatty and amino acid composition and lower concentrations of total fat and vitamin D and B12. On the other hand, research shows that for some nutrients, for example choline, no differences in the milk concentration between women following a vegan or omnivorous diet was found, and Pawlak et al. reported reduced levels of vitamin B12 in milk from both vegan and omnivorous women (7–9). However, the influence of a vegan diet on vitamin B2 and carnitine concentrations in human milk is largely unknown and needs to be elucidated as this could have consequences for the breastfed infant as well.

Vitamin B2, also called riboflavin, is a water-soluble vitamin that needs to be consumed through the diet as it is not endogenously synthesized. Vitamin B2 is mainly present in animal food sources, including dairy products, meat and fish and to a lesser extent in some fruits and vegetables (10). Vitamin B2 is involved in many important biological processes, including redox homeostasis, DNA repair, protein folding, and bioenergetics (11, 12). Hence, a shortage in vitamin B2 concentrations could have detrimental metabolic consequences. There is evidence that vitamin B2 deficiency in infants can lead to anemia, neurologic abnormalities, and visual impairment (10). Carnitine is a conditionally essential nutrient as in a omnivorous diet about 25% is synthesized by the body (13), while the other 75% has to be provided via the diet. Carnitine is mostly present in meat, fish, and dairy products, but can to a lesser extent be found in plant-based products (14). Carnitine is essential for the transfer of activated long-chain fatty acids into the mitochondria destined for β-oxidation, and is therefore important in energy production from fat (15, 16). Symptoms of a carnitine deficiency in infants include for example hypoglycaemia, (cardio) myopathy, and encephalopathy (15, 17).

In general, individuals following a vegan diet have an increased risk of low serum vitamin B2 and carnitine concentrations (1–6). Lactating women have a higher nutrient requirement due to milk production. It can be hypothesized that lactating women following a vegan diet may have an even higher risk of developing a vitamin B2 and carnitine deficiency with subsequently lower concentrations of vitamin B2 and carnitine in their serum and milk compared to milk derived from omnivorous women. To investigate this we compared the vitamin B2 and carnitine concentrations in milk and serum of lactating women following a vegan and an omnivorous diet.

## 2. Methods

### 2.1. Study design

The participants for the current study were selected out of over 2,000 lactating women who participated in the COVID MILK—POWER MILK cohort study, which was conducted between October 2020 and February 2021 (18). For the study, women during any lactation stage were recruited if they were willing to once donate a small portion of expressed milk and if a maternal blood sample could be collected. Besides, an extensive questionnaire was filled out by all participants, including information on their diet. The study was approved by the Ethics Committee of the Amsterdam University Medical Center, VUmc (NL74752.029.20) and written informed consent to use their samples for future research was obtained from all participants.

### 2.2. Matching process and selection of study participants

**Figure 1:**
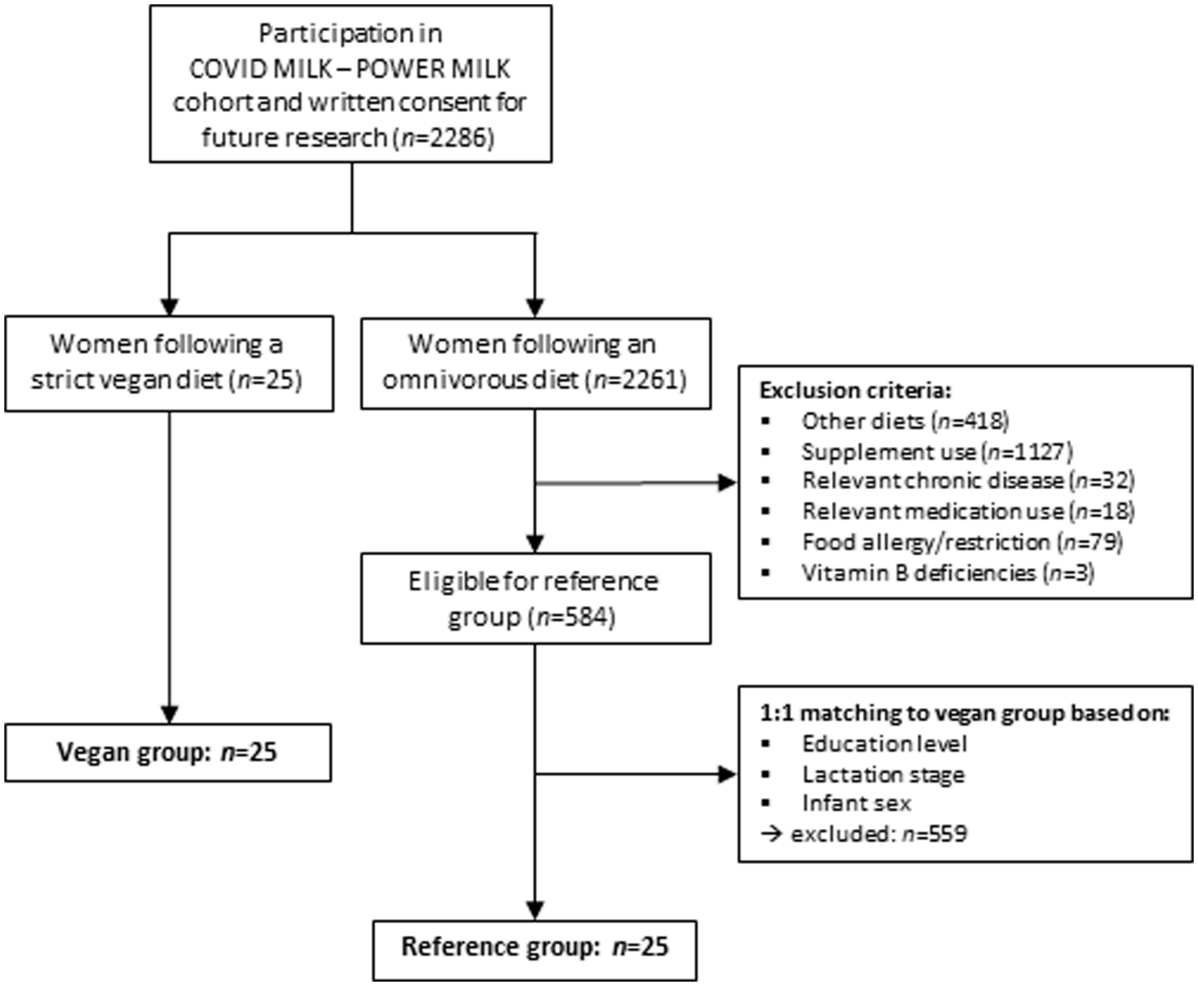
Flowchart and matching process.

From the total COVID MILK—POWER MILK cohort, participants were excluded that did not provide permission to use their samples for other research than measuring SARS-CoV-2 immunoglobulins. Among the remaining participants, 25 lactating women followed a strict vegan diet and were eligible to be included in the vegan group of the current study. To compare vitamin B2 and carnitine concentrations in human milk and serum, an omnivorous reference group was matched to these 25 vegan participants. Exclusion criteria for the reference group were (1) following another diet, including vegetarian, flexitarian, macrobiotic, lactose-free, egg-free, anthroposophical, gluten-free, and religious-related diets (*n* = 418), (2) nutritional supplement use (*n* = 1,127), (3) chronic diseases or medication use that might influence vitamin B2 or carnitine uptake or concentrations (*n* = 50), (4) food allergies that could influence their dietary intake (*n* = 79), or (5) other known vitamin B deficiencies (anamnestic; *n* = 3). After applying these exclusion criteria, 584 participants were eligible for inclusion in the reference group. From these 584 participants, 25 participants were matched 1:1 to the participants in the vegan group, based on lactation stage and educational level. These matching criteria were selected as it has been demonstrated that they are associated with vitamin B2 and carnitine concentrations (19–22). The flowchart of the matching process is depicted in Figure 1. Case–control matching in IBM SPSS statistics 26 for Windows was used for the matching process. Matching margins were set at ±30 days for lactation stage and at the same educational level.

### 2.3. Sample collection

Human milk samples were collected in the morning, before the first feeding moment of the infant. For the sample collection, the whole breast was emptied. The participant was requested to mix the milk, and to donate 10–30 mL in a sterile container. Participants stored the milk samples in their refrigerator up until collection by the researcher after which the milk samples were stored in different aliquots at the Amsterdam UMC at −80°C until analyses. A serum sample of 5 mL was collected by a phlebotomist at the same date of the milk donation and stored in different aliquots at −80°C until analyses.

### 2.4. Laboratory analyses

Riboflavin = vitamin B2 was analyzed by UPLC with fluorimetric detection, essentially as described by Capo-Chichi et al. (23). Briefly, 100 μL serum/milk plus 20 μL of galactoflavin (internal standard, final concentration 10 nM) was deproteinized using 50 μL of 1 N perchloric acid, incubated for 30 min at 4°C, and centrifuged at 14.000 × *g* for 10 min. Ten microliter of the supernatant was injected onto a Ultimate 3000 UPLC system (Thermo Scientific), and vitamin B2 was detected fluorimetrically (excitation 450 nm, emission 530 nm). Plasma (acetyl)carnitines were determined by flow injection MS/MS as described previously (24). In brief, the following internal standards were added to 50 μL of serum/milk: 1,250 pmol 2H3-carnitine (for free carnitine) and 250 pmol 2H3-propionylcarnitine (for acetylcarnitine) and deptroteinized using 500 μL of acetonitril while vortexing. After centrifugation, the supernatant was taken to dryness under a nitrogen flow and the residue derivatized using 100 μL butylation reagent (butanol/acetylchoride 4:1 v/v) for 15 min at 60°C, taken to dryness under nitrogen flow, and resuspended in 50 μL acetonitril. This was injected into a Xevo TQ-S MICRO system (Waters) in the positive ion mode. Neolynx was used to quantify carnitine and acetylcarnitine using the internal standards.

### 2.5. Statistical analyses

Baseline characteristics were described using descriptive statistics including frequencies, mean with standard deviation (SD), or median with interquartile range (IQR, reported as Q1-Q3). Differences in baseline characteristics including maternal current BMI, age, and lactation stage were tested, to identify possible differences between the groups that might influence the results of this study. The statistical tests performed to test the baseline characteristics were according to the nature of the variable and distribution. Normality of the data was determined by visually inspecting histograms and Q-Q plots of the data. For variables that were normally distributed, an independent *t*-test was performed, for data not normally distributed, a non-parametric Mann Whitney U test was performed, and for frequencies, a chi-square test was performed. To determine differences in vitamin B2 and carnitine concentrations between study groups, a multiple regression was performed with the “reference group” treated as reference, taking into account characteristics that differed between study groups. Milk and serum concentrations were log-transformed prior to the regression analyses. To correct for this logarithmic transformation and to aid interpretation of the outcomes, the following formulas were used to accurately interpret the outcomes: Regression coefficient = e^β and 95.0% confidence interval = $e^{\left(\beta \pm 1.96\;x\;standarderror\right)}$ . In case of a missing sample, the sample of the matched case was also excluded. No data imputation was conducted. A value of *p* smaller than 0.05 was used to determine significance. For carnitine, both free carnitine (C_0_) and acetylcarnitine (C_2_) were included as outcomes in the analyses. Statistical analyses were performed using IBM SPSS statistics 26 for Windows. GraphPad Prism 9 for Windows was used to illustrate the findings in graphs.

## 3. Results

Baseline characteristics are shown in Table 1 and did not differ between study groups, except for maternal current BMI (*p* = 0.013), which was lower in the vegan group. The majority of the participants in the vegan group took nutritional supplements (92%), of which 57% contained vitamin B2. Women in the vegan group had a median daily vitamin B2 intake through supplements of 0.7 mg (IQR 0–2.1). None of the participants used carnitine supplements. All participating women reported to not have changed their diets recently or during their pregnancy. Table 2 shows the median (IQR) values of vitamin B2 and carnitine in human milk and serum for the vegan and the reference group. A multiple regression model, with BMI as a covariate in the adjusted model, was used to determine differences between study groups. Vitamin B2 concentrations in human milk and serum did not differ between study groups. Moreover, whereas there were no difference in human milk carnitine concentrations between study groups, the carnitine serum concentrations were lower in participants following a vegan diet, also after adjustment of BMI.

**Table 1:** Participants’ baseline characteristics (*n* = 25 in each group).

Characteristics	Total	Vegan	Reference	*p value*
Age mother (years)	33 (31–35)	33 (31–36)	34 (31–35)	0.83
*median* (*IQR*)				
Current Body Mass Index (kg/m^2^)	23.2 (21.6–27.2)	22.2 (20.6–26.4)	24.2 (23.0–29.2)	0.01^*^
*median* (*IQR*)				
Educational level ISCED
Low education—*n* (%)	2 (4)	1 (4)	1 (4)	
Medium education—*n* (%)	4 (8)	2 (8)	2 (8)	
High education—*n* (%)	44 (88)	22 (88)	22 (88)	
Lactation stage (days)	290 (187–431)	305 (200–434)	273 (155–437)	0.76
*median* (*IQR*)				
Gender of child				0.57
Female—*n* (*%*)	26 (52)	14 (56)	12 (48)	
Male—*n* (*%*)	24 (48)	11 (44)	13 (52)	
Supplement use – *n* (*%*)				
Containing Vit B2—*n* (*%*) Intake per day (mg)^1^	23 (46)	23 (92)	0 (0)	
	-	13 (57)	-	
	-	0.7 (0–2.1)	-	
*median* (*IQR*)				
Parity				0.30
First child—*n* (*%*)	25 (50)	14 (56)	11 (44)	
Second child—*n* (*%*)	17 (34)	9 (36)	8 (32)	
Third child—*n* (*%*)	8 (16)	2 (8)	6 (24)	

IQR, interquartile range; Education is classified according to the ISCED (International Standard Classification of Education ISCED; *p<0.05).

**Table 2:** Vitamin B2 and carnitine (C_0_ and C_2_) concentrations in human milk per study groups.

	Human milk				Serum			
	Vegan group	Reference group	Crude *β* (*95% CI*)	Adjusted *β* (*95% CI*)	Vegan group	Refernce group	Crude *β* (*95% CI*)	Adjusted *β* (*95% CI*)
Vitamin B2 (nmol/L) *median* (*IQR*)	137.0 (42.0–310.5)	139.0 (104.5–241.5)	0.89 (0.67–1.18)	0.91 (0.68–1.22)	19.0 (16.0–29.0)	16.0 (13.5–31.0)	0.99 (0.81–1.20)	0.99 (0.81–1.22)
Free carnitine (C_0_) (μmol/L) *median* (*IQR*)	39.0 (30.4–54.9)	39.7 (34.0–52.3)	0.98 (0.90–1.07)	0.97 (0.89–1.07)	27.2 (23.0–30.8)	37.8 (33.0–43.0)	0.86 (0.82–0.90)^****^	0.87 (0.83–0.92)^****^
Acetylcarnitine (C_2_) (μmol/L) *median* (*IQR*)	8.2 (6.9–15.3)	9.9 (7.7–14.6)	0.99 (0.83–1.18)	0.97 (0.81–1.17)	3.7 (3.3–4.4)	4.7 (3.9–6.8)	0.88 (0.82–0.94)^***^	0.87 (0.81–0.94)^****^

Differences between study groups are determined using a multiple regression model with log-transformed values. After analysis, the values are corrected for the performed logarithmic transformation.

β = regression coefficient (corrected for performed logarithmic transformation).

95.0% CI = 95.0% confidence interval (corrected for performed logarithmic transformation).

Adjusted analysis [Adjusted β (95% CI)] is adjusted for maternal BMI.

^***^p < 0.001, ^****^p < 0.0001.

Human milk samples: *n* = 50, Serum samples: *n* = 48.

**Figure 2:**
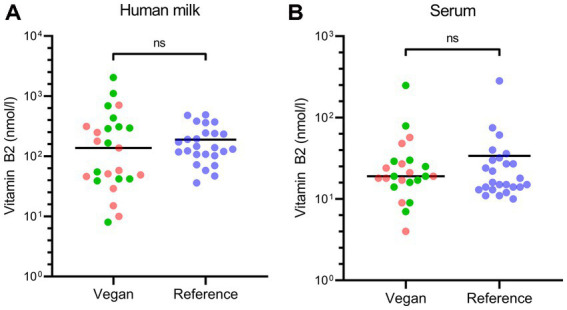
Vitamin B2 concentrations in human milk and serum for each study group. This figure shows the individual human milk (panel **A**) and serum (panel **B**) concentrations of vitamin B2 in the vegan and reference groups. The lines represent the median concentrations of the study groups. In the vegan group, participants indicated by a red dot did not take vitamin B2 supplements; participants indicated by a green dot did take vitamin B2 supplements. Ns, not significant.

**Figure 3:**
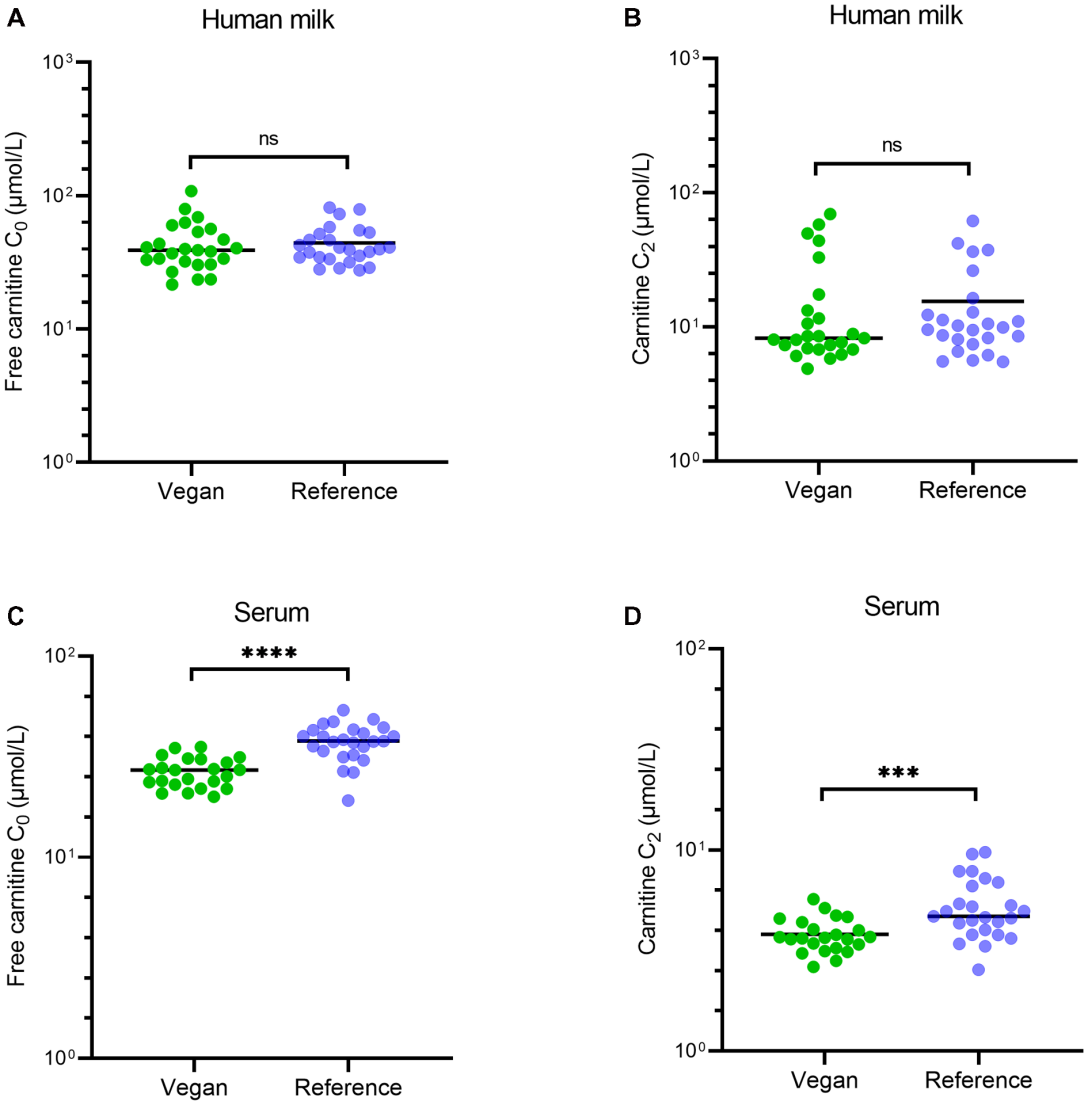
Carnitine (C_0_ and C_2_) concentrations in human milk and serum for each study group. This figure shows the individual human milk concentrations for free carnitine C_0_ (panel **A**) and for acetyl carnitine C_2_ (panel **B**) of both study groups. Panel **(C)** shows the serum concentrations of free carnitine C_0_, and panel **(D)** shows the serum concentrations of acetyl carnitine C_2_ of both study groups. The lines represent the median concentrations of the study groups. Ns, not significant, ^****^indicates a value of *p* < 0.0001, ^***^indicates a value of *p* < 0.001.

The individual vitamin B2 concentrations for each study group are visualized in Figure 2 and the carnitine concentrations in Figure 3. There were no differences in human milk vitamin B2 concentrations between participants who used vitamin B2 supplements and participants who did not (*p* = 0.43).

## 4. Discussion

In this study, we aimed to investigate the influence of a vegan diet on the vitamin B2 and carnitine concentrations in milk and serum of lactating women. Vitamin B2 concentrations did not differ between study groups in both milk and serum. While participants following a vegan diet had lower serum carnitine concentrations compared to participants with an omnivorous diet, they had similar human milk carnitine concentrations.

Although previous research on the effects of a vegan diet on vitamin B2 concentrations in human milk is lacking, it has been demonstrated that individuals following a vegan diet have lower serum concentrations of vitamin B2 (1, 3–6, 25, 26). In contrast to prior research on vitamin B2 serum concentrations and our hypothesis on milk concentrations, we did not find differences in vitamin B2 concentrations in milk or serum between study groups. One of the possible explanations for this difference is that half of the participants following a vegan diet used nutritional supplements with a high amount of vitamin B2, whereas none of the participants in the reference group used supplements. However, we found no differences in human milk vitamin B2 concentrations between participants with or without intake from supplements. The median intake from vitamin B2 supplements was 0.7 mg per day in the vegan study group, which is 44% of the daily recommended intake of 1.6 mg for lactating women (27). Also, as our study population was overall highly educated and given that socioeconomic status is associated with the quality of dietary intake (19), it could be suggested that the participants in our study compensated their intake for possible nutrient shortages in their diet.

The lower serum carnitine concentrations in participants following a vegan diet are in line with previous research (28). One study investigated the effect of dietary carnitine intake on carnitine concentrations in human milk, and found, similar to our results, no association between maternal intake and human milk carnitine (29). It is known that Na^+^-dependent L-carnitine carriers in the mammary gland actively transport carnitine into the milk, independent of the serum concentration (30). The fact that human milk concentrations remain stable while the serum concentrations are decreased could be explained by active transport of carnitine into the milk to sustain carnitine supplementation to the child.

Studying the effect of a vegan diet on human milk composition is important as it might influence the nutritional value of milk and subsequently growth and development of the infant. As we did not find differences in milk vitamin B2 and carnitine concentrations between lactating women following either a strict vegan or omnivorous diet, we do not expect that a maternal vegan diet cause a vitamin B2 or carnitine deficiency in the breastfed infant. Carnitine concentrations in human milk of lactating mothers following a vegan diet were similar compared to the reference group. However, maternal serum carnitine concentrations were on average below the normal serum carnitine range in women (31, 32). Although infants seem to receive appropriate amounts of carnitine when the mothers consume a vegan diet, for the mother herself it could be considered to increase her carnitine intake through diet or supplements.

This study has some limitations. First, information on the maternal diet and supplemental intake was obtained via an online questionnaire, which could have led to self-reporting bias. Additionally, we investigated the effect of an overall diet, not actual intake, on human milk concentrations. The actual intake of mothers following a vegan diet differs from person to person. However, we assume that our participants likely represent average vegan lactating women, limiting the effect of dietary variation on our results. Second, our sample size was relatively small, as a result of which we could have missed small differences between study groups and it should be mentioned that results need to be interpreted with caution. Third, serum samples were not specifically collected in the fasting state, which may have an influence on the measurement of nutrients. Fourth, women in our cohort had a median lactation duration of 9 months. Normally at this stage, infants receive complementary feeding and human milk is not the sole source of carnitine and vitamin B2 anymore. Moreover, we did not measure the complete nutritional profile of human milk, as consuming a vegan diet could also influence other important nutrients. Finally, we did not have data to investigate the direct effects on the infant. Future, large sample-sized studies are needed to address this question. Our study was strengthened by our standardized human milk sample collection. Furthermore, participants were matched 1:1 from a large cohort, which limits the effect of other factors on our outcomes, for example lactating stage. Additionally, we investigated both human milk and serum concentrations, enabling us to observe differences between these biofluids.

## 5. Conclusion

Our study indicates that vitamin B2 and carnitine concentrations in human milk are not affected by consumption of a vegan diet. Serum carnitine concentrations, however, were lower in vegan lactating women suggesting active compensatory transport of carnitine into milk. These results suggest that a vegan diet in lactating mothers is not a risk for the development of a vitamin B2 or carnitine deficiency in breastfed infants. This information is useful for donor human milk banks, which collect milk for provision to premature infants who do not receive sufficient mother’s own milk.

## Data availability statement

The raw data supporting the conclusions of this article will be made available by the authors, without undue reservation.

## Ethics statement

The studies involving human participants were reviewed and approved by METc VU Medisch Center. The patients/participants provided their written informed consent to participate in this study.

## Author contributions

HJ, CA, FV, JG, and BK designed the research. HJ, PM, FV, and BK conducted the research and analyzed data or performed statistical analysis. AK and JG provided essential materials. HJ, CA, PM, AK, FV, JD, and BK wrote the paper. JG and BK had primary responsibility for final content. All authors contributed to the article and approved the submitted version.

## Funding

This research was funded by Stichting Steun Emma Kinderziekenhuis. This funding source had no role in the design of this study and did not have any role during its execution, analyses, interpretation of the data, or decision to submit results.

## Conflict of interest

JG is founder and director of the Dutch National Human Milk Bank and member of the National Health Council, and has been a member of the National Breastfeeding Council from March 2010 to March 2020.

The remaining authors declare that the research was conducted in the absence of any commercial or financial relationships that could be construed as a potential conflict of interest.

## Publisher’s note

All claims expressed in this article are solely those of the authors and do not necessarily represent those of their affiliated organizations, or those of the publisher, the editors and the reviewers. Any product that may be evaluated in this article, or claim that may be made by its manufacturer, is not guaranteed or endorsed by the publisher.
